# On the Structure of the Membrana Tympani in the Human Ear

**Published:** 1850-11

**Authors:** 


*On the Structure of the Membrana Tympani in the Human Ear.*—Mr. Joseph Toynbee, in a paper read at the Royal Society on the 20th June, describes the Membrana Tympani as consisting of the following layers, quite distinct from each other, both as regards their structure and functions.

1. Epidermis.
2. The proper fibrous layer, composed of
   *a.* The lamina of radiating fibres.
   *b.* The lamina of circular fibres.
3. Mucous membrane.

One of the principal objects of the paper is to describe the structure and functions of the fibrous laminæ. Since the time of Sir Everard Home, who pronounced the layer of radiating fibres to be muscular, anatomists have differed in their views of the nature of the fibrous element of the Membrana Tympani. The lamina of radiating fibres, the outer surface of which is covered by the epidermis, is continuous with the periosteum of the external meatus. With the exception of the uppermost fibres, which, on account of their being somewhat flaccid, have been considered as a separate tissue, under the name of " membrana flaccida," the radiate layer is composed of fibres which extend from the circular cartilaginous ring to the malleus, and they interlace in their course. These fibres are from the 4000th to the 5000th part of an inch in breadth.

The lamina of circular fibres consists of fibres, which are firm and strong towards the circumference, but very attenuated towards the centre. These fibres are so attached and arranged, as to form a layer of membrane, which, in a quiescent state is saucer-shaped. The fibres composing the circular are smaller than those of the radiate lamina being from the 6000th to the 10,000th part of an inch in breadth.

The facts that appear to be adverse to the idea of the fibres of either layer being muscular, are :—

1. The absence of distinct nuclei in the fibres.
2. Their great denseness and hardness.

The four laminæ forming the Membrana Tympani are continuous with the other structures, of which they appear to be mere modifications, and not one is proper to the organ.

The tensor tympani ligament, which had not been previously noticed by anatomists, is particularly described by Mr. Toynbee in the paper read before the Royal Society. It is attached externally to the malleus, close to the insertion of the tensor tympani muscle, and internally to the cochleariform process.

The latter part of the paper is occupied by observations on the functions of the fibrous laminæ, and of the tensor ligament of the Membrana Tympani; and it is shown that by these two antagonistic forces, the one tending to draw the Membrana Tympani outwards, the other inwards, this organ is maintained in a state of moderate tension, and is always in a condition to receive ordinary sonorous undulations.—*London Journal of Medicine.*

